# Thermal and Optical Properties of the Sunspace Casing as Factors Influencing Temperature Rise in Greenhouse Systems

**DOI:** 10.3390/ma14237411

**Published:** 2021-12-03

**Authors:** Magdalena Grudzińska

**Affiliations:** Faculty of Civil Engineering and Architecture, Lublin University of Technology, 40 Nadbystrzycka Str., 20-618 Lublin, Poland; m.grudzinska@pollub.pl

**Keywords:** sunspaces, temperature reduction factor, dynamic simulations, multifamily buildings

## Abstract

In sunspaces, there is an observable temperature rise above the external air temperature, caused by solar gains and the buffering effect of their enclosure. In addition, their external partitions form a barrier preventing the direct influence of the external environment and delaying the natural deterioration of elevation surface. In the paper, the temperature rise in a glazed balcony attached to a typical flat in a multifamily building, together with the energy demand in the living zone, were assessed with the use of dynamic computer simulations. Ten variants of the sunspace casing were analysed, with different thermal and solar energy transmittance of the glazing (which is a novel subject in the research area). This enabled us to evaluate average values of the temperature reduction factor during the year and to choose the most efficient variant of the sunspace external partitions. It turned out to be an insulated, double-glazed casing with a spectrally selective coating (type O 21), combining high insulative properties with high solar transmittance. These features allowed the temperature in the sunspace to rise by almost 10°C (compared with the external air) and lower total energy demand in the flat by 33% (compared with a flat with an open balcony).

## 1. Introduction and Literature Review

The phenomenon of temperature increase in rooms enclosed with transparent partitions was already known in ancient times. The first mentions of greenhouses and winter gardens come from Rome and Greece and date back to the beginning of the common era [1]. Originally, greenhouses were used in agriculture and horticulture to accelerate plant growth or the cultivation of species requiring temperatures higher than those found in a given climate zone. Since then, orangeries have established themselves in European architecture. They were used for both practical purposes and as a sign of the financial status of the owners. By the end of the nineteenth century, the combination of glazed spaces with living space was initiated. "Solar rooms" were generally adjacent to the living rooms on the southern side and were used for periodic heating of the interiors. In addition to performing practical functions, they were also a place of integration with nature and the natural environment, often constituting original and attractive architectural elements [2].

By the end of the twentieth century, global energy crises became the driving force behind renewed interest in the use of solar radiation and other renewable energy sources in construction and architecture. Currently, sunspaces in the form of built-up balconies or loggias can be seen more and more often in newly built and existing buildings in urban areas. They perform various functions, such as [3,4,5]:

- Practical functions, providing additional recreational or economic space, as well as increasing the sense of security of users;
- Factors related to energy saving, owing to the reduction in heat loss from adjacent rooms and preheating the air used for their ventilation;
- Factors related to the protection of residential units from noise, which is particularly important in the areas with high traffic;
- Factors related to the protection of the façade and rooms of the building from weather conditions, such as pollution, precipitation, wind, and temperature fluctuations.

Building a solar space and increasing the temperature on the outer side of the partitions surrounding the heated zone of flats is a very beneficial phenomenon from the point of view of building operating costs. First of all, it reduces heat losses and energy consumption for central heating and also reduces the risk of moisture condensation in the places of thermal bridges. In conjunction with the protection of the façade against weather conditions, it increases the durability of its enclosure. Solutions of this type can be classified as structural methods of protection against the natural destruction of building materials and partitions.

Research works (both experimental and analytical) on greenhouse systems clearly show an increase in temperature above the ambient temperature in a glazed, unheated space, which is a primary result of the solar operation. This effect was reported for all kinds of building structures, starting from small-scale research chambers [6] to single- and multi-family buildings.

Extensive temperature monitoring in multifamily buildings was carried out by Hilliaho and colleagues in 2009–2010 in 22 apartments (17 of which had glazed balconies) located in Tampere [7]. The recorded temperature was higher on all enclosed balconies than on open balconies, and the average increase was approximately 5 °C above the outside air temperature [8]. Balconies’ glazing was made of single uncoated panes (plain glass) with gaps of several millimeters between the panes. If the cladding of the solar space is more airtight, the average temperature increase on balconies during winter can be up to about 15 °C. Such results have been achieved in the case of balcony enclosures made of double glazing in PVC profiles in multifamily houses located in Bucharest [9]. Similar results in the Polish climate were obtained by Grudzińska, who recorded an average temperature increase from about 3 °C to 8 °C in balconies with leaky enclosures and about 15 °C in areas of a tightly built loggia [10]. The monitored flats were located in multifamily buildings in Lublin and Zamość, cities in the eastern part of Poland.

Greenhouse systems in single-family buildings are of lesser importance for protection against heat loss and protection of the façade because glazed outbuildings occupy a smaller part thereof. Nevertheless, in such facilities, a significant temperature increase in the greenhouse was also observed by Rempel et al. [11]. During research conducted in Japan by Ma et al., temperatures of up to 40 °C were recorded on sunny winter days inside the glazed loggia, and the period in which temperature exceeded 30 °C accounted for over 70% of the measurement time from November to February [12,13]. Experimental studies on historic small buildings in Spain also confirmed the effectiveness of greenhouses in passive heating of the air. As described in [14,15], the average temperature rise in the glazed balcony during autumn and winter amounted to almost 3 °C. These analyses concerned not only the favourable phenomena in winter, but also addressed the problem of ensuring comfortable conditions in living quarters in summer, owing to the combination of passive solar energy acquisition with heat accumulation through massive building partitions [16,17]. The negative effects of solar radiation conversion in the countries with a warm climate, such as decreased thermal comfort or increased demand for cooling, were also presented in other works [18,19].

The temperature rise in a greenhouse influences energy demand in the adjacent rooms. When sunspaces are combined with the building’s ventilation system, it is possible to transport the preheated air into living spaces, which can be of great relevance for energy saving. The authors of [20] studied the possibilities of reducing heating demand in a building equipped with a modular sunspace prototype placed on the roof, reaching a 25% to 58% reduction in energy use (depending on the climatic conditions). Ma et al. [12] experimentally investigated the thermal performance of a sunspace attached to a house with a central air conditioning system, distributing the preheated air from the solar space. The process was activated depending on the temperature difference between the adjacent bedroom and the sunspace. The results showed that approximate energy savings reached 12% compared to a house without a sunspace. Using a sunspace for preheating the air before it entering the HVAC equipment was also studied in [21]. Two configurations were examined there: collecting the energy during the whole day or only at night, taking advantage of the sunspace thermal inertia, and the first one turned to be more effective from the economical point of view. A publication by Ulpiani et al. [22] analysed the influence of additional sunspace glazing on the internal temperature and energy gains, connecting it with irradiative (without air transport between sunspace and living space) or convective (with air transport) use. The convective double-glazed configuration was found to outperform the others, reducing the energy use by more than 27% for all weather conditions. 

A different approach, also giving good results as to the energy savings, was using the buffer effect of the solar space, without air exchange with the living zone. This strategy allowed higher internal temperatures to be obtained within the sunspace, and some researchers reported this solution to be more effective than an intensive air extraction [13].

An important issue in improving the solar energy conversion by the sunspace was incorporating some elements that increase the thermal capacity of its envelope. A parametric study using the validated simulation model was conducted to examine, among others, the effects of rock-bed thermal storage on greenhouse functioning [23]. The paper [24] investigated detached residential buildings with a sunspace and a concrete thermal storage wall, either containing or not containing a phase-change material (PCM). The analysis of annual energy requirements of the building models revealed heating and cooling energy use reduction in the most profitable PCM configuration reaching about 2%, compared with a reference model with no PCM. The effects of a PCM louver integrated with a sunspace on the energy consumption for heating a rural residence in China were analysed numerically within the study [25]. This solution allowed to lower the energy demand by approximately 14% at the most and reduce energy consumption fluctuations compared with a traditional sunspace without such louvers. 

The described research points out that the choice of building materials, elements, and airflow intensity in the sunspace enclosure may have a noticeable impact on the temperature rise and the effectiveness of the solar energy conversion. However, there is a research gap in this area, considering the influence of the optical properties of glazing on the internal conditions in the solar space. In most of the up-to-date research, plain glazing was used in the construction of the sunspace envelope. Occasionally, glazing described as "low-e" was adopted, without more detailed reference to its optical and thermal properties [22,26,27,28,29,30]. The knowledge on the subject was partially widened by the paper [31], describing the effects of using sunspace on heating energy consumption of a rural house in connection with the sunspace glazing type, the filling gas type, and the gas layer thickness. The results showed that energy savings of approximately 19% and 33% could be attained by using single and double glazing, respectively. They also allowed for making conclusions on the optimal thickness of the gases filling the space between the panes. 

Nevertheless, the subject of coatings modifying the optical and thermal properties of the glazing in the context of sunspaces is still lacking comprehensive studies. Technical development in this field prompts the extension of the existing analyses and consideration of the possibility of regulating the heat balance, owing to spectrally selective coatings. These coatings are applied on glass panes during or after the production process and enable adjustment of the transmittance and reflectance of radiation in specific ranges of solar radiation. The modifications generally aim at keeping the transmittance of visible radiation at a high level, while lowering the transmittance (and thus increasing the reflectance) in the near- and far-infrared spectrum. It helps to protect the glazed interiors from overheating and to diminish the radiative heat losses to the outside [32]. 

The presented paper is intended to fill this gap by extensive analyses based on validated computer simulations of an exemplary flat in a multifamily building, located in warm-summer humid continental climate conditions. Ten variants of the sunspace casing will be taken into account, with different thermal and solar energy transmittance of the glazing, allowing the regulation of internal conditions in the greenhouse. The positive effect of the temperature rise will be evaluated by the temperature reduction factor, depending on the temperature difference between the heated living area, unheated sunspace, and the air outside. Thanks to the analyses it will be also possible to assess energy demand in the flat throughout the year, and to choose the most efficient variant of the sunspace external partitions.

## 2. Materials and Methods

The temperature of the glazed space depends on the combined effects of solar gain, enclosure heat loss, infiltration, and user behaviour. It can convey information about the dominant methods of heat transfer and the magnitude of the greenhouse effect [18]. However, the temperature time series itself is difficult to apply in more precise comparative studies due to its high variability over time as well as dependence on the instantaneous values of external temperature and insolation. A quantitative measure in this range can be the temperature correction factor *b_tr_* between the heated room, the sunspace, and the outside environment. It depends on the temperature increase in the greenhouse above the outside air temperature, i.e., it reflects the buffer effect of the non-air-conditioned glazed space and the possibility of reducing heat loss through the partition between the greenhouse and the heated zone. It is related to the physical characteristics of the greenhouse enclosure, such as thermal insulation, the ability to absorb, transmit, and reflect radiation, as well as the tightness of the external partitions and user behaviour related to, e.g., ventilation of sunspace or the use of sunshades. This relatively simple indicator is also necessary to calculate the energy demand in a heated space adjacent to the solar space based on the monthly method in the standard [33], allowing us to include sunspace solar gains during heating or cooling seasons [34]. 

The temperature correction factor is calculated as [35]:

(1) $$b_{tr}=\frac{\theta_{i}-\theta_{s}}{\theta_{i}-\theta_{e}}$$

where:

*θ_i_*—mean internal temperature in the living room adjacent to the balcony in a given calculation step [°C];
*θ_e_*—mean outside temperature in a given calculation step [°C];
*θs*—mean internal temperature in the solar space in a given calculation step [°C].

**Figure 1:**
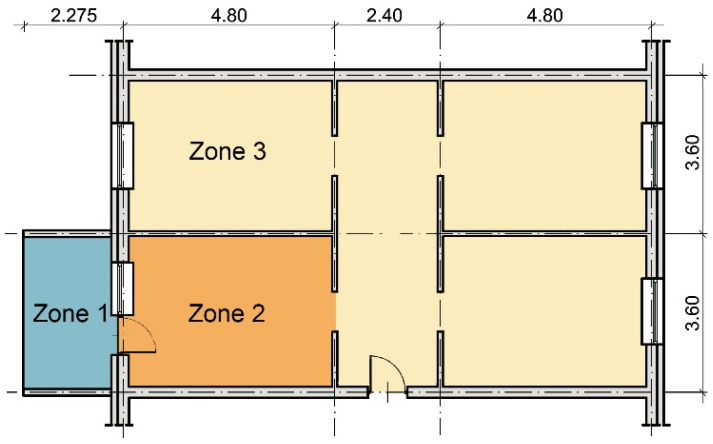
The modelled apartment with thermal zones.

Numerical computer simulations with an hourly step were used to determine the course of temperature in the rooms. The simulations were performed in the BSim programme, based on the control volume method. It modelled an exemplary apartment (with an area of 75 m^2^) in a multifamily building, located in the central part of the repetitive storey. It had two opposite external walls, and the other walls and ceilings separate them from rooms with a similar function. The rooms were arranged symmetrically, with windows in opposite walls. One of the rooms was adjacent to an unheated balcony with an area of approx. 5 m^2^, accessible through a door in an external partition wall (Figure 1). Thermal insulation of the external partitions of the apartment met the requirements in force in Poland since 2014 (external walls: U ≤ 0.25 W/(m^2^·K), roofs: U ≤ 0.20 W/(m^2^·K), windows: U ≤ 1.30 W/(m^2^·K)).

The model distinguished three thermal zones—a balcony, an adjacent room, and other living quarters, for which internal conditions and the air exchange with the environment were defined in different ways. The balcony was an unheated zone. The residential area was air-conditioned while the assumed heating and cooling settings were 20 °C and 26 °C. Internal living gains in the apartment were assumed in accordance with [36] at the level of 7.10 W/m^2^. The ventilation air in zone 2 was preheated in the balcony area, and in zone 3 it came directly from the outside.

**Figure 2:**
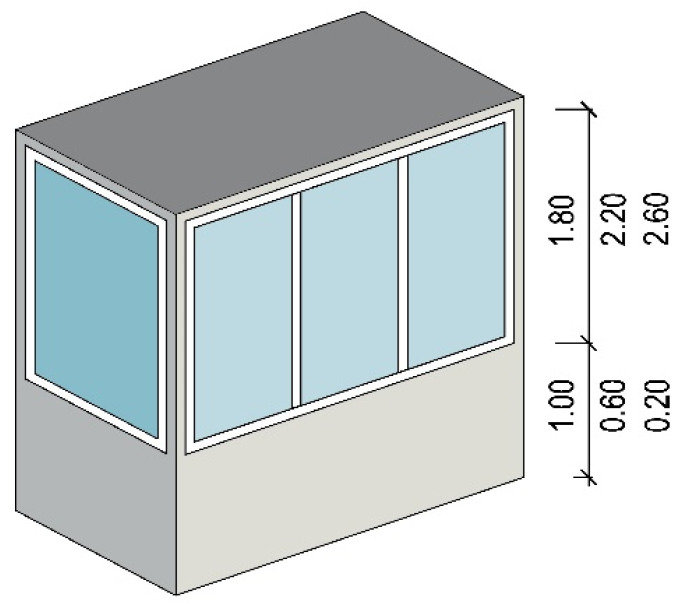
Glazing height and layout.

The balcony enclosure was glazed on all external walls, which were 2.80 m high. The height of the glazing was assumed to be equal to 1.80 m, 2.20 m, or 2.60 m, in combination with a wall below windows with a height of 1.00, 0.60, or 0.20 m, respectively (Figure 2). 

The enclosure itself could be characterised by low or high thermal insulation (Table 1). This corresponds to the solutions most often used in multifamily buildings, which represent two types of operation of passive systems:

- Oriented towards maximising solar gains owing to glazing with low thermal insulation and high solar radiation transmittance;
- Oriented at minimising heat losses by increasing thermal insulation, generally associated with a reduction in solar gains.

**Figure 3:**
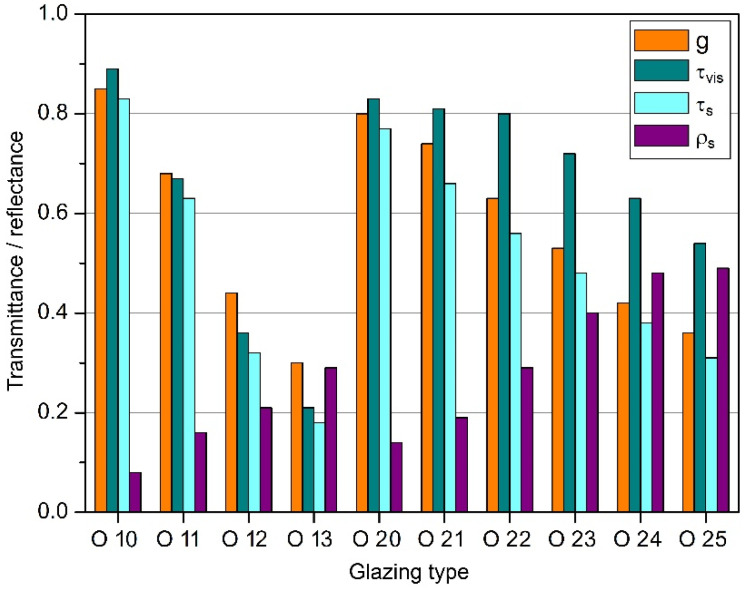
Optical parameters of glazing (based on [37]).

Single-pane glazing was used in the enclosure with low insulation, mounted in a frameless system (O 10 to O 13), and in the enclosure with intermediate and high insulation, double-glazed glazing (O 20 to O 25) was used. Sets marked from O 10 to O 13 were single-pane sets made of 8 mm thick tempered glass (Table 1). The basic glazing was O 10, made of ordinary glass without spectrally selective coatings, characterised by the highest transmittance of radiation but also the lowest thermal insulation. The remaining single-pane glazing had a coating on the inside, which reduced the transmittance of solar energy and visible radiation. Due to the greater ability to reflect infrared radiation, the O 12 and O 13 sets had slightly better thermal insulation. The sets marked from O 20 to O 25 were double-pane 4/12/4, sets, with the interpane space filled with argon or krypton (Table 1). The O 20 set was made of plane glass, and the others had spectrally selective coatings applied on one or two panes (O 24 and O 25). Increasing the reflectivity of infrared radiation increased their thermal insulation, but this was at the expense of reducing solar energy transmittance (Figure 3).

To maximise passive gains, the balcony glazing was closed and exposed during the winter period. During the transitional period, it was possible to ventilate the balcony by opening the glazing, if the internal air temperature exceeded 22 °C. It was also assumed that overheating could be reduced by using curtains with a radiation transmittance of 50% if the internal temperature exceeded 24 °C or the intensity of solar radiation transmitted through the glazing exceeded 300 W/m^2^. The thresholds of ventilation and shading in residential premises were assumed at the level of 24 °C and 25 °C, respectively.

Taking into account changes in individual parameters, a total of 150 calculation cases, described in Table 2, were analysed.

## 3. Validation of the Simulation Programme

**Figure 4:**
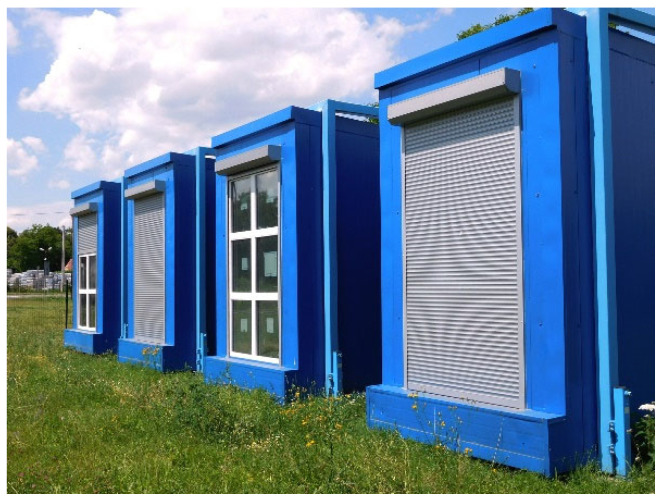
Climatic chambers, Rzeszów University of Technology.

The simulation programme was validated based on measurements carried out in climatic chambers of the Rzeszów University of Technology [38]. They were designed to conduct research on passive solar systems under the actual climatic conditions (Figure 4). 

**Figure 5:**
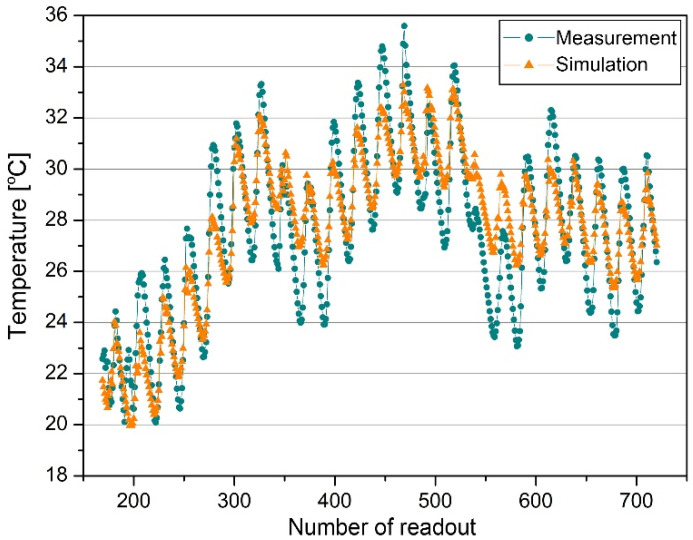
Temperature time series coming from measurements and simulations, 18 May 2016–9 June 2016.

The measurements carried out from 11 May 2016 to 9 June 2016 were used for validation. During this period, the chamber was not air-conditioned, but the internal air temperature, the temperature on the surface of full and glazed partitions as well as heat fluxes flowing through the glazing were recorded at intervals of 10 minutes. The following parameters of the external environment were measured at the same time intervals: outside air temperature and humidity, pressure, wind direction and speed, solar radiation intensity on the horizontal plane. The air temperature obtained from the computer model was compared with the temperature measured in the chamber in the validation process (Figure 5).

Based on the measurements and simulations, the following statistical parameters were determined (Table 3):

- Mean Bias Error (*MBE*)
  (2) $$MBE=\frac{\sum_{i=1}^{n}\left(S_{i}-M_{i}\right)}{\sum_{i=1}^{n}M_{i}}\cdot 100\%$$
- Root-Mean-Square Error (*RMSE*)
  (3) $$RMSE=\sqrt{\frac{\sum_{i=1}^{n}{\left(S_{i}-M_{i}\right)}^{2}}{n}}$$
- Coefficient of variation of Root-Mean-Square Error (*Cv(RMSE)*)
  (4) $$Cv\left(RMSE\right)=\frac{\sqrt{\frac{\sum_{i=1}^{n}{\left(S_{i}-M_{i}\right)}^{2}}{n}}}{\bar{M}}\cdot 100\%$$
- Mean Absolute Percentage Error (*MAPE*)
  (5) $$MAPE=\frac{\sum_{i=1}^{n}\left|\frac{S_{i}-M_{i}}{M_{i}}\right|}{n}\cdot 100\%$$

where:

*M*—measured value;
*M’*—mean measured value;
*S*—value determined based on simulation;
*n*—number of observations.

Recommendations for the models subjected to calibration were presented in studies [39,40,41], and the boundary values for the particular statistical parameters were gathered in Table 4.

Using the above-mentioned criteria, the presented model could be assessed positively, as both the *MBE* and *Cv(RMSE)* values calculated for the measured and simulated chamber temperature were far below the level recommended by the American Society of Heating, Refrigerating and Air-Conditioning Engineers (ASHRAE) [39], the International Performance Measurement and Verification Protocol Committee (IPMVP) [40], and the Federal Energy Management Program [41]. Moreover, the MAPE value did not exceed 5%, which, according to [42], allowed the model to be assessed as good.

## 4. Climatic Data Used in the Simulation Model

The simulations used climatic data from the Typical Meteorological Year (TMY) for the Warszawa Okęcie station (latitude 52°10′ E, longitude 20°58′ N, the height of the station 107 m a.s.l.). Warsaw is often taken as representative of Poland’s climate because the air temperature and sunshine conditions there correspond to the average values for the entire country.

According to TMY, the lowest average temperature of outside air occurred in January, February, and December −1.2 °C, −0.9 °C, and 0.8 °C, respectively), whereas the highest were in June, July, and August (17.1 °C, 19.2 °C, 16.6 °C). The extremes of temperature were 12.3 °C (January) and 33.2 °C (August). The smallest insolation occurred in December, November, and January, while the highest was in July, June, and May. The diffuse component had the dominant share in total radiation, which on average accounted for 76% in the heating season (September to May) and 65% in the remaining months.

**Figure 6:**
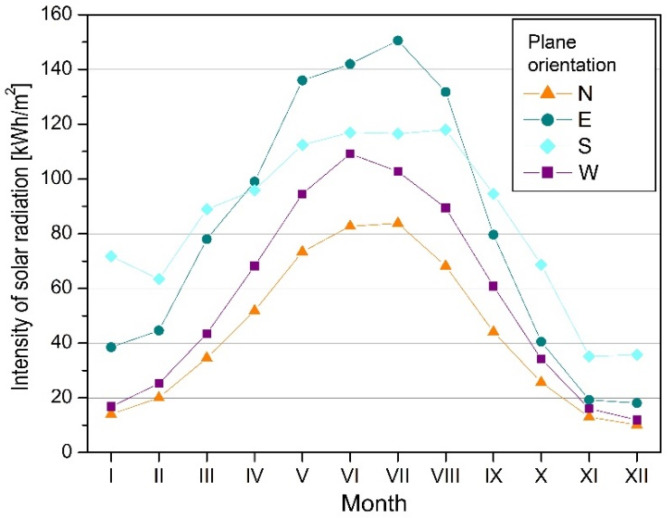
Solar radiation on vertical planes, calculated according to Perez model.

Obtaining energy through the greenhouse system would depend to a large extent on the intensity of radiation reaching the vertical planes with different orientations to the directions of the world (Figure 6). These values were determined in the simulations using the Perez model [43], which showed good compliance with the results of measurements carried out in Poland [44]. The calculations also took into account the radiation reflected from the ground surface. The greatest amounts of solar radiation reached vertical planes directed to the southeast and south (Table 5, Figure 6) from September to May (during the heating season). The planes directed to the north and northwest were characterised by the least insolation during the year.

## 5. Results and Discussion

### 5.1. Temperature Rise in the Sunspce and the Temperature Correction Factor

**Figure 7:**
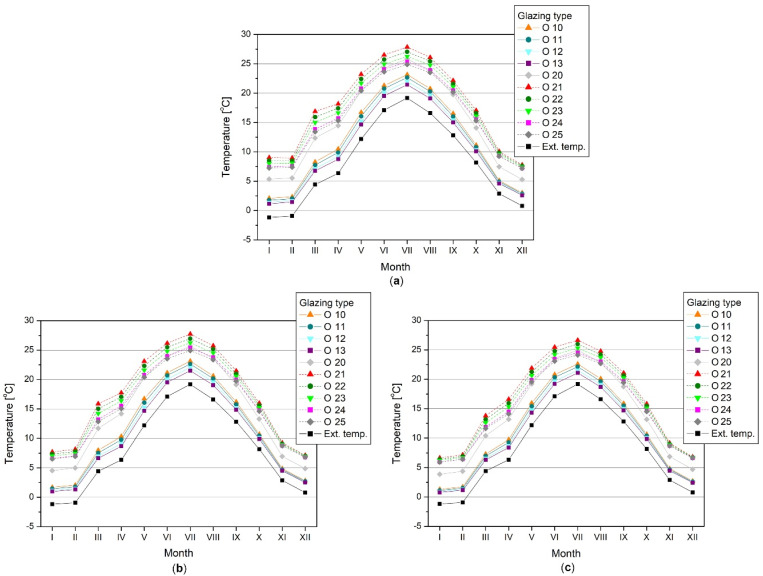
Monthly averaged sunspace temperature (zone 1), glazing height 2.20 m: (**a**) balcony facing south; (**b**) balcony facing east; (**c**) balcony facing west.

Based on the simulation, the average monthly sunspace temperatures for all glazing variants turned out to be higher than the outside air temperature (Figure 7), ranging from −1.2 °C in January to 19.2 °C in July. The temperature rise in the greenhouse followed the external temperature time series and was greater for insulated enclosures with double glazing, averaging from about 6.5 °C (west-facing balcony, O 25 glazing) to around 9.6 °C (south-facing balcony, O 21 glazing). In the case of uninsulated casings with single glazing, the temperature increase ranged from about 1.9° C to 3.5 °C (for a balcony facing west, glazing type O 13 and balcony facing south, glazing type O 10, respectively). The higher the total solar energy transmittance of the glazing (g), the higher the internal sunspace temperature (regarding the specific type of the envelope).

Considering the balcony’s orientation, directing it towards the south allowed the temperature rise to be higher, up to 0.8 °C and 2.5 °C compared with the least favourable west direction (for uninsulated casings with single glazing and insulated casings with double glazing, respectively).

The dependence of temperature on the glazing height was slight, regardless of the balcony orientation and glazing type, and the differences between the extreme cases did not exceed 0.2 °C (uninsulated casings with single glazing) and 0.3 °C (insulated casings with double glazing). Increasing the glazing area increased solar gains, but also increased heat losses through panes with lower thermal insulation than a full enclosure, so the resulting greenhouse temperature was comparable. Therefore, the remainder of the results are presented only for an intermediate glazing height of 2.20 m.

The regularities that could be observed in the temperature time series were as follows:

- The greatest efficiency in converting solar radiation was demonstrated by insulated enclosures, with the coating allowing the highest solar radiation transmittance through glazed parts;
- The lowest effectiveness was observed for uninsulated enclosures, with glazing and coatings characterised by the lowest radiation transmittance;
- The temperature increase was greatest if the balcony faced south and southeast, and the west orientation was the least favourable in this respect, which resulted from the shape of the enclosure and the distribution of radiation on vertical planes with different orientations (Table 5);
- The greatest increase in temperature in the glazed space took place from May to August, and in the case of insulated enclosures with higher radiation transmittance, the average temperature of zone one (balcony) may be up to 3 °C higher than the temperature of zone two (living room),
- November and December were characterised by the lowest efficiency of obtaining solar energy by greenhouse systems and the lowest temperature increase, which resulted from the lowest supply of solar radiation and the lowest irradiation of vertical planes.

**Figure 8:**
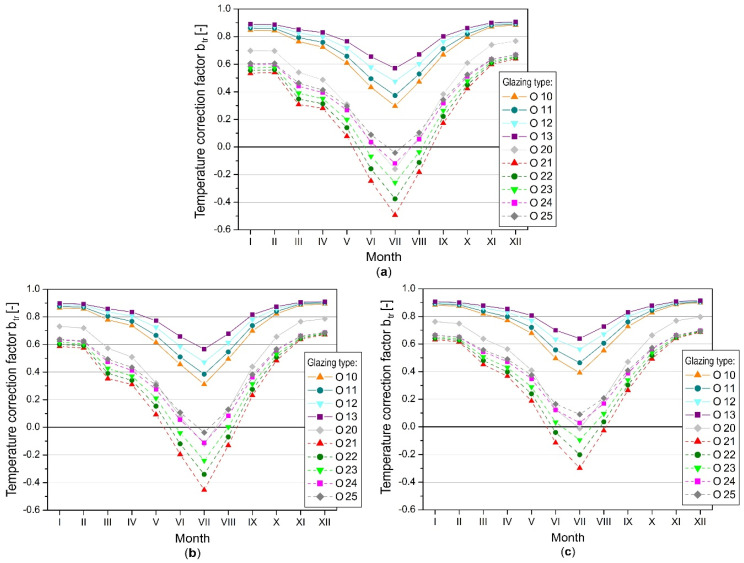
Temperature correction factor, glazing height 2.20 m: (**a**) balcony facing south; (**b**) balcony facing east; (**c**) balcony facing west.

The temperature-correction factor was determined based on the monthly mean temperatures in zones two and one, and the graphs are presented for individual types of glazing and selected balcony orientations (Figure 8). 

The b_tr_ course was similar in all cases. Temperature-correction factors peaked in December and then declined until July. From November to February, the coefficients were almost constant; deviations from the maximum value did not exceed 15%. The highest b_tr_ values meant that the temperature difference between the living room and the sunspace was large, which took place during the winter. These differences decreased in the summer and transitional periods, which resulted in a decrease in the b_tr_ coefficient. The temperature correction factor was negative if the temperature in the balcony area was higher than in the living room, which in some cases occurred between May and September.

The influence of the type of glazing and the type of enclosure on the ability to regulate the temperature was visible, and the graphs for sunspaces with single and double glazing were clearly separated. The lowest ability to increase the temperature in the greenhouse volume was found in uninsulated casings with single glazing. 

The b_tr_ coefficients were the highest for these solutions, which meant they had the lowest buffer effect of the greenhouse and the highest heat losses from the heated room (zone 2). Intermediate and insulated casings, with more airtight double glazing, created buffer zones that use solar gains more efficiently to increase the temperature inside the greenhouse. The lower solar gains in the balcony area, caused by the lower permeability of the glazing, were compensated by lower heat losses to the environment, allowing for higher temperatures than in the case of enclosures with low thermal insulation and greater solar radiation transmission capacity.

It was also observed that in both groups of enclosures (uninsulated and insulated), the greater the radiation transmittance through the glazing, the smaller the temperature correction factors, i.e., the higher the temperatures in the glazed space. The moderately insulated enclosure with uncoated double glazing (O 20) deviated to some extent from these trends. Despite the slight difference between the g-factors of O 20 and O 21 glazing, the b_tr_ was much lower for the latter. This was due to the greater dissipation of heat to the environment through the enclosure with O 20 glazing, with a higher heat loss coefficient.

During the heating season, the south and southeast direction allowed for the smallest temperature correction factors, and the heat demand itself in zone two was also the lowest. The highest coefficients, as well as the highest heat demand in the room adjacent to the balcony, were determined for the western orientation (Figure 8c).

### 5.2. Energy Demand in the Heated Area

**Figure 9:**
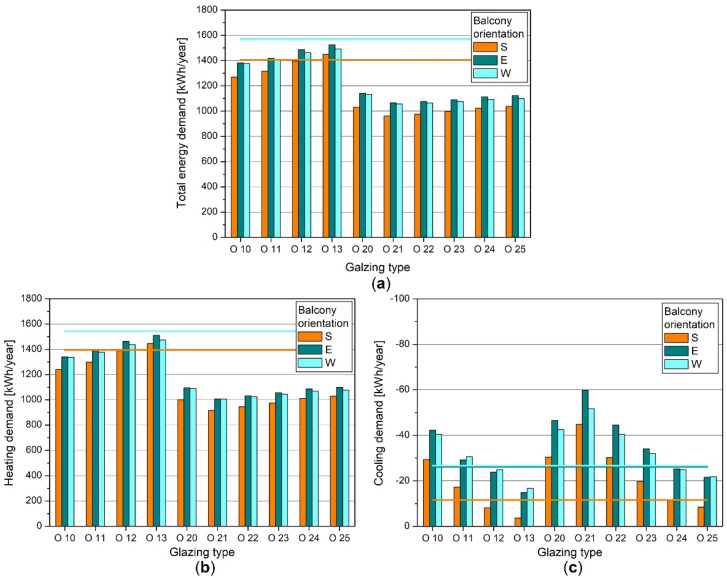
Energy demand in the flat, glazing height 2.20 m: (**a**) total energy demand; (**b**) heating demand; (**c**) cooling demand. Bars depict energy demand in the apartments with glazed balconies, lines—in the apartment with an open balcony.

The noted temperature rise in the area of the glazed balcony had a consequences in changing the energy demand of the heated living area during the whole year (Figure 9).

In the heating season, the biggest energy savings (compared with the flat with an open balcony) could be seen for the insulated envelopes with double glazing. They amounted from approximately 28% to 35%, reaching maximum values for the glazing type O 21. In the case of the uninsulated envelope, the best solution was the glazing O 10, giving energy savings of 10–12%, and the worst was O 13, causing a rise in heating needs of up to 4% if the balcony is facing south. The reason for this was the restriction of solar gains in zone two due to small solar energy transmittance (g = 0.30). 

Heating demand was the smallest if the balcony was facing south or southeast. The least profitable orientations were the eastern and southwestern ones, and the results obtained for the western orientation were similar in most cases (Figure 9b). Changes caused by the rotation of the flat from the southern direction amounted from 5% to 9% (uninsulated casings with single glazing) and from 8% to 10% (insulated casings with double glazing).

Analysing cooling demand, the glazings with total solar energy transmittance (g) lower than 0.5 allowed reduced the energy need below the level specified for the flat with an open balcony (Figure 9c). This applied both for the insulated and uninsulated casings and the glazing types O 1.2, O 1.3, O 2.4, O 2.5. In the remaining cases, enclosing the balcony increased cooling demand up to four times, considering double glazing with the biggest height (2.60 m).

The south orientation was the most favourable from the point of view of the cooling needs, thanks to the smallest energy gains in the northern part of the apartment. Other directions gave much higher energy demand, which could rise by 35% to 430% (uninsulated casings with single glazing) or by 26% to 208% (insulated casings with double glazing).

Total energy demand (being the sum of heating and cooling needs) showed similar trends to heating demand, as the Polish climate is oriented toward heating the buildings rather than cooling. In almost all cases, the positive effects of the balconies’ enclosure during the colder part of the year outweighed the drawbacks occurring during summer, and the total energy savings reached up to 33% compared with the flat with an open balcony. The most favourable orientation turned out to be the southern one, thanks to the smallest components of the energy balance during the heating and cooling season.

Among all of the analysed glazing types, the best solution was the insulated encasement with double glazing O 21, regardless of the glazing height or the balconies’ orientation. These results were consistent with the analyses of the b_tr_ factor, also indicating this configuration as giving the biggest potential of energy savings thanks to the highest temperature within the sunspace and the lowest values of the temperature-correction factor. 

## 6. Conclusions

The implementation of glazed solar spaces in residential buildings may constitute a type of structural protection of the façade against weather conditions and natural degradation processes. Increasing the external temperature also has a positive effect on reducing the risk of moisture condensation in the places of thermal bridges and on the internal surfaces of partitions, delaying the aging and corrosion processes of building materials.

The effectiveness of the protections themselves is clearly dependent on the selection of materials used in the construction of the enclosure—primarily on their thermal insulation and (in the case of transparent elements) the ability to transmit short-wave solar radiation and reflect long-wave thermal radiation. These factors turned out to have the greatest impact on the efficiency of radiation conversion and the possibility of increasing the temperature in the greenhouse space.

In the presented research, the thermal and optical properties of glazing and balconies’ envelopement and their influence on the temperature in the sunspace and energy demand in the living space were given thorough consideration. The key findings of the work may be summarised as follows:

- Evaluation of the performance of glazed balconies as passive solar systems with the use of the temperature-correction factor proved to be consistent with the energy needs analyses of the conditioned apartment; this demonstrates the usefulness of the b_tr_ coefficient as a measure of solar conversion potential of a sunspace;
- There was a visible difference in the functioning of the passive greenhouse system and its ability to convert solar radiation into heat energy, depending on the insulating properties of the sunspace envelope and the total solar energy transmittance of the glazing;
- Envelopes with added thermal insulation and double glazing were clearly more effective solar collectors compared with uninsulated casing and single glazing; this emphasised the importance of the buffer effect provided by the sunspace, and the advantage of the strategy depending on minimising heat losses over maximizing solar gains in the greenhouse;
- High effectiveness of solar conversion during the heating season may raise overheating risk during summer, but because in the warm-summer humid continental climate conditions building maintenance is oriented towards heating rather than cooling, these effects turned out to be negligible when taking into account the whole-year energy needs;
- Changes in the solar energy transmittance turned out to be a useful solution helping to influence temperature rise in the sunspace and the energy demand in the apartment; the most effective variant of the envelope was an insulated, double-glazed casing with the spectrally selective coating (type O 21); it combined high insulative properties with high solar transmittance, allowing to raise the temperature in the sunspace by almost 10 °C (compared with the external air) and lower total energy demand in the flat by 33% (compared with the flat with an open balcony).

The paper is a part of a broader analysis of the energy efficiency of greenhouse systems in multifamily buildings located in the continental climate of Poland. In connection with the topic of building façade protection, further studies on the impact of glazed balconies on the heat and moisture flow at the joints of structural elements and potential thermal bridges are planned.

## Figures and Tables

**Table 1:** Configuration of glazing and balcony enclosure type.

Glazing Type	O 10	O 11	O 12	O 13	O 20	O 21	O 22	O 23	O 24	O 25
**No of Panes**	1	1	1	1	2	2	2	2	2	2
**Selective Coating**	−	+	+	+	−	+	+	+	+	+
**U_g_** **[W/(m^2^·K)]**	5.60	5.60	5.30	5.10	2.70	1.40	1.30	1.20	1.10	1.00
**g** **[–]**	0.85	0.68	0.44	0.30	0.80	0.74	0.63	0.53	0.42	0.36
**τ_vis_** **[–]**	0.89	0.67	0.36	0.21	0.83	0.81	0.8	0.72	0.63	0.54
**τ_s_** **[–]**	0.83	0.63	0.32	0.18	0.77	0.66	0.56	0.48	0.38	0.31
**ρ_s_** **[–]**	0.08	0.16	0.21	0.29	0.14	0.19	0.29	0.4	0.48	0.49
**g/U_g_** **[(m^2^·K)/W]**	0.15	0.12	0.08	0.06	0.30	0.53	0.48	0.44	0.38	0.36
**Window Frame**	Alu	Alu	Alu	Alu	PVC	PVC	PVC	PVC	PVC	PVC
**U_f_** **[W/(m^2^·K)]**	6.00	6.00	6.00	6.00	2.00	1.20	1.20	1.20	1.20	1.20
**U_w_** **[W/(m^2^·K)]**	5.70	5.70	5.40	5.30	2.60	1.40	1.30	1.20	1.10	1.00
**Opaque Enclosure**	NI	NI	NI	NI	IT	I	I	I	I	I
**U_e_** **[W/(m^2^·K)]**	1.60	1.60	1.60	1.60	0.65	0.30	0.30	0.30	0.30	0.30

U_g_—thermal transmittance of the glazing; g—total solar energy transmittance; τ_vis_—visible radiation transmittance (380 nm–780 nm); τ_s_—solar radiation transmittance (300 nm–2500 nm); ρ_s_—solar radiation reflectance (300 nm–2500 nm); U_f_—thermal transmittance of the window frame; U_w_—thermal transmittance of the window; U_e_—thermal transmittance of the opaque part of the enclosure. Opaque enclosure: NI—not insulated, IT—intermediate, I—insulated.

**Table 2:** The range of the basic parameters assumed in the simulations.

Parameter	No of Variants	Range
Building location	1	Warsaw
Balcony orientation	5	S, SE, E, SW, W
Apartment location	1	Central part of the repetitive storey
Thermal insulation of the external partitions	1	0.25 W/(m^2^·K)/1.30 W/(m^2^·K) (walls/windows)
Air change	2	0.5 1/h/0.5 1/h/1.4 1/h (zone 3/2/1) Balcony—not insulated 0.5 1/h/0.3 1/h/0.8 1/h (zone 3/2/1) Balcony—intermediate or insulated
Balcony type	1	External, enclosure gazed on 3 walls
Balcony enclosure	3	Not insulated, intermediate or insulated
Glazing type	10	Described in Table 2
Glazing height	3	1.80 m, 2.20 m, 2.60 m
Absorptivity of balcony enclosure	1	0.5

**Table 3:** Statistical parameters for the data from 18 May 2016–9 June 2016.

*MBE* [%]	*RMSE* [°C]	*CV(RMSE)* [%]	*MAPE* [%]
0.57	1.44	5.22	4.38

**Table 4:** Calibration criteria for the simulation models using hourly data.

Statistical Parameter	Publication		
	ASHRAE 14 [39]	IPMVP [40]	FEMP [41]
*MBE* [%]	±10	±5	±10
*Cv(RMSE)* [%]	30	20	30

**Table 5:** Solar radiation intensity on the vertical planes of different orientations (blue marks the lowest sums and yellow marks the highest).

Time Period	Solar Radiation Intensity on the Planes of Different Orientations [kWh/m^2^]							
	N	NE	E	SE	S	SW	W	NW
I	14.0	15.3	38.5	70.1	71.7	40.8	16.8	14.0
II	20.0	24.9	44.6	64.5	63.4	41.9	25.2	20.0
III	34.6	48.3	78.0	98.1	88.9	62.3	43.5	35.2
IV	51.8	73.0	99.0	107.6	95.9	82.5	68.2	54.8
V	73.4	107.3	136.0	134.0	112.4	105.6	94.5	77.1
VI	82.7	116.2	142.0	136.5	117.0	117.2	109.2	89.0
VII	83.8	123.2	150.6	141.6	116.5	112.4	102.7	84.5
VIII	68.2	101.0	131.8	135.6	118.0	106.4	89.4	70.3
IX	44.1	57.5	79.6	95.6	94.5	79.7	60.8	46.6
X	25.6	27.6	40.5	60.9	68.7	53.7	34.2	25.9
XI	13.0	13.3	19.2	30.8	35.2	26.7	16.1	13.0
XII	10.1	10.2	18.1	32.6	35.7	23.8	11.9	10.1
IX–V	286.6	377.4	553.5	694.2	666.4	517.0	371.2	296.7
Whole year	521.3	717.8	977.9	1107.9	1017.9	853.0	672.5	540.5
